# Association between monocyte-to-high-density lipoprotein cholesterol ratio and stroke: a propensity score matched cross-sectional study

**DOI:** 10.3389/fneur.2026.1721994

**Published:** 2026-04-16

**Authors:** Qing Huang, Yuanyuan Li

**Affiliations:** 1 Department of Neurology, Shulan (Quzhou) Hospital, Quzhou, Zhejiang, China; 2 Department of Endocrinology, Shulan (Quzhou) Hospital, Quzhou, Zhejiang, China

**Keywords:** stroke, monocyte-to-high-density lipoprotein cholesterol ratio, National Health and Nutrition Examination Survey, propensity score matching, restricted cubic spline, cross-sectional study

## Abstract

**Background:**

Stroke is one of the leading causes of death and long-term disability worldwide, and chronic inflammation plays a central role in its pathogenesis. The monocyte-to-high-density lipoprotein cholesterol ratio (MHR) integrates pro-inflammatory activity and anti-atherosclerotic capacity, but its association with stroke risk in the general population remains unclear. This study aims to explore the relationship between MHR and stroke among U.S. adults.

**Methods:**

In this cross-sectional study, we leveraged data from the 1999–2020 National Health and Nutrition Examination Survey (NHANES). Multivariable logistic regression models were employed to examine the relationship between the MHR and stroke risk. Restricted cubic spline (RCS) analysis assessed potential non-linear dose–response relationships, while subgroup analyses evaluated its robustness. Propensity score matching (PSM) was applied to minimize confounding bias through sample refinement.

**Results:**

The analytical cohort encompassed 43,321 participants, including 1,583 individuals with physician-diagnosed stroke. Following PSM, the non-stroke comparator group was refined to 4,719 subjects. In the fully adjusted multivariable model, elevated MHR demonstrated a statistically significant association with stroke predisposition [Odds ratio (OR) = 1.517; 95% Confidence interval (CI):1.096–2.099]. RCS regression confirmed a linear dose–response gradient between MHR and stroke risk (*P*-non-linear = 0.9517). Stratified subgroup analyses further validated the robustness of this relationship across demographic and clinical strata (all *P* for interaction >0.05), indicating consistent effect magnitudes.

**Conclusion:**

In this nationally representative cross-sectional study, higher MHR levels were associated with an increased risk of stroke. Although these findings suggest that MHR may be a potential biomarker related to stroke risk, further prospective studies are required to validate its temporal relationship and clinical applicability.

## Introduction

Stroke is the second leading cause of death and the third leading cause of long-term disability globally, imposing a heavy burden on more than 7 million individuals each year (1). This growing disease burden has made stroke a key issue in global public health. Notably, recent epidemiological evidence indicates a rising incidence of stroke among younger adults (2), underscoring the urgent need for early risk identification. Therefore, identifying novel and independent biomarkers among adults aged 20 and older could aid in early detection strategies and promote more targeted stroke prevention efforts.

Increasing evidence suggests that systemic inflammation plays a crucial role in the development and progression of atherosclerosis, significantly raising the risk of stroke through various mechanisms (3). Monocytes, as key coordinators of vascular inflammation, infiltrate atherosclerotic lesions and differentiate into macrophages. By secreting pro-inflammatory cytokines and matrix-degrading enzymes, they drive plaque formation, progression, and instability (4), playing a pathogenic role in the onset and progression of stroke (5). In contrast, high-density lipoprotein cholesterol (HDL-C) exerts an inhibitory effect on atherosclerosis by promoting reverse cholesterol transport and exhibiting anti-inflammatory and antioxidant properties (6). Studies have shown that HDL-C can protect endothelial cells from inflammatory and oxidative stress by preventing monocyte recruitment to the arterial wall and regulating monocyte activation and precursor cell proliferation (7). Therefore, a composite indicator reflecting the balance between monocyte-mediated inflammation and HDL-C’s anti-inflammatory capabilities may offer a biologically meaningful tool for assessing the risk of ischemic stroke.

Previous studies have observed an association between the monocyte-to-HDL cholesterol ratio (MHR) and adverse clinical outcomes in various cardiovascular diseases (8–10). However, the relationship between MHR and stroke risk in large population cohorts has not been explored. Capitalizing on this unaddressed research void, our investigation leverages the nationally representative National Health and Nutrition Examination Survey (NHANES) database to elucidate the relationship between the MHR and stroke susceptibility.

## Materials and methods

### Study design and participants

**Figure 1:**
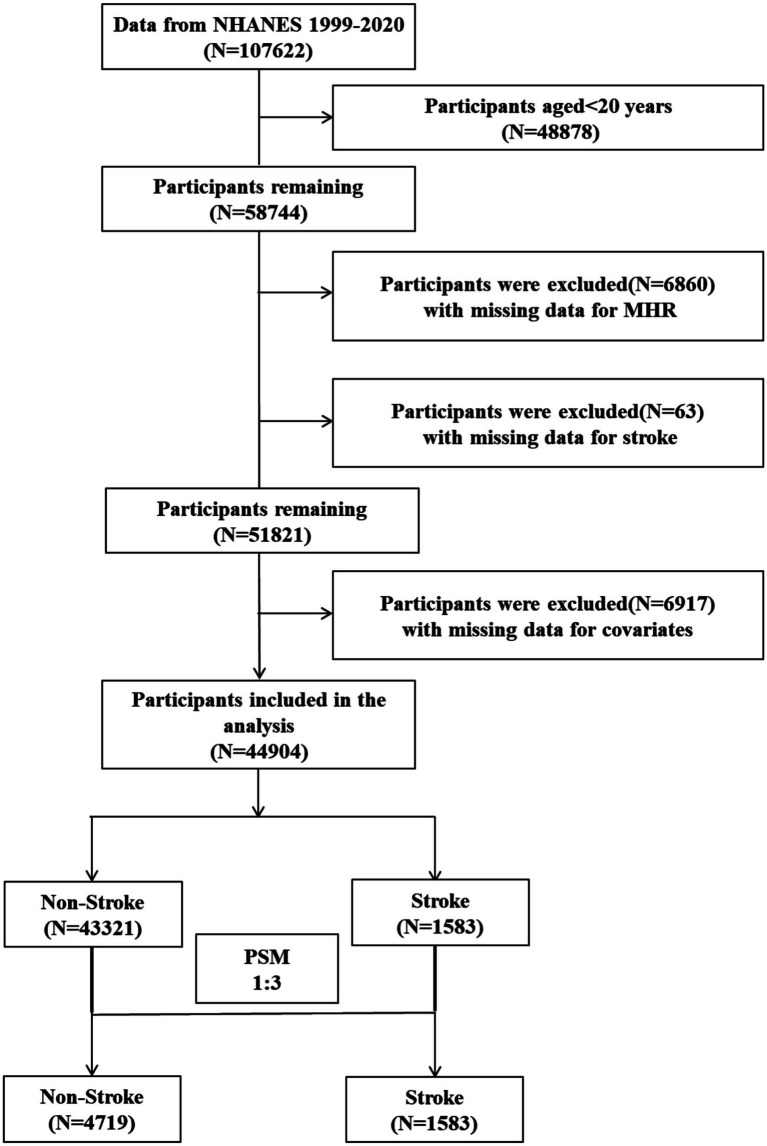
Flow chart of participant selection. MHR, monocyte-to-high-density lipoprotein cholesterol ratio; PSM, propensity score matching; NHANES, National Health and Nutrition Examination Survey.

This cross-sectional investigation utilized data from the 1999–2020 NHANES cycles. NHANES is a nationally representative cross-sectional survey aimed at assessing the health and nutritional status of the U.S. non-institutionalized population. NHANES combines standardized interviews, physical examinations, and laboratory measurements to collect comprehensive demographic, socioeconomic, lifestyle, clinical, and biochemical health information. The initial cohort comprised 107,622 participants. After sequential exclusions of individuals aged <20 years (*n* = 48,878), those with missing MHR values (*n* = 6,860), and subjects lacking stroke diagnosis records (*n* = 63), the analytical sample included 44,904 eligible adults: 43,321 non-stroke controls and 1,583 stroke cases. Propensity score matching (PSM) at 1:3 ratio generated a refined matched cohort of 4,719 controls and 1,583 stroke patients. The participant selection cascade is depicted in Figure 1.

### Measurement of MHR

The MHR was derived by dividing absolute monocyte counts (expressed in 10^3^ cells/μL) by serum HDL-C concentrations (measured in mmol/L). All hematological specimens—including monocyte enumeration and HDL-C quantification—were processed using Beckman Coulter MAXM analyzers at Mobile Examination Centers (MECs), following standardized protocols (11).


$$\mathrm{MHR}=\frac{\text{Monocyte Count}\;(10^{9}/L)}{\mathrm{HDL}-C\;\text{Concentration}\;(\text{mmol}/L)}$$

### Stroke outcomes

Stroke diagnosis relied on retrospectively collected self-reported medical histories. Participants were systematically queried: Has a physician or other healthcare professional ever diagnosed you with stroke? Affirmative responses classified individuals as stroke cases. This definition includes both ischemic and hemorrhagic strokes and has been widely adopted in previous NHANES-based epidemiological studies. We explicitly acknowledged the potential for retrospective recall inaccuracies inherent in self-reported data, which may introduce measurement bias affecting outcome validity and clinical interpretation (12).

### Covariates

Covariates were pre-selected based on established clinical relevance, prior epidemiological evidence, and the availability of data from the NHANES survey cycles (13, 14). These covariates included demographic characteristics (age, sex, race), socioeconomic indicators [education status, ratio of family income to poverty (PIR), Marital status], physical activity levels (PA), body mass index (BMI), cardiovascular and metabolic comorbidities [diabetes, hypertension, coronary heart disease (CHD)], and laboratory biomarkers reflecting liver and kidney function [alanine aminotransferase (ALT), aspartate aminotransferase (AST), and serum creatinine (Scr)]. Detailed definitions, coding schemes, and inclusion criteria for all covariates are provided in Supplementary Table S1.

### Statistical analysis

To accurately reflect the complex sampling design of NHANES and obtain nationally representative estimates, all analyses were conducted using survey weights in accordance with the analytic guidelines.^1^ MEC examination weights were standardized according to the survey cycle of each participant as follows: for the 1999–2002 cycles, the weight was calculated as (WTMEC4YR × 4)/21.2; for the 2003–2016 cycles, the weight was calculated as (WTMEC2YR × 2)/21.2; and for the 2017–2020 cycle, the weight was calculated as (WTMECPRP × 3.2)/21.2. Baseline characteristic disparities between stroke and non-stroke cohorts were assessed using Student’s *t*-tests for continuous variables and *χ*^2^ tests for categorical measures, with continuous parameters presented as weighted means ± standard deviations and categorical variables reported as unweighted frequencies (weight-adjusted percentages).

To reduce confounding, PSM was performed. The propensity score was estimated using a logistic regression model that included all covariates except the primary exposure variable in the multivariable analysis. PSM was implemented via a 1:3 nearest-neighbor algorithm with a caliper width of 0.1 to balance cohort characteristics. Covariate balance before and after PSM was assessed using absolute standardized mean differences (SMD), with an SMD below 0.1 indicating minimal residual imbalance.

A multivariable logistic regression model was employed to assess the association between MHR and stroke, with results presented as odds ratios (OR) and 95% confidence intervals (CI). Model 1 was unadjusted; Model 2 adjusted for age, sex, race, BMI, education status, marital status, PIR and PA; Model 3 further adjusted for diabetes, hypertension, CHD, AST, ALT, and Scr. Restricted cubic spline (RCS) with four knots evaluated potential nonlinear relationships, while stratified subgroup analyses explored latent effect modifiers across demographic and clinical domains. All statistical procedures were executed in R (version 4.2.2), with two-tailed *p* < 0.05 defining statistical significance.

## Results

### Patient demographics and baseline characteristics

Among 44,904 participants, 1,583 were diagnosed with stroke. Within this cohort, the stroke group exhibited significantly elevated MHR compared to non-stroke counterparts, alongside higher mean age (63.864 ± 14.298 years), greater female predominance (57.86%), predominant non-Hispanic White ethnicity (72.33%), lower educational attainment, increased body mass index (BMI: 29.862 ± 6.935 kg/m^2^), and heightened comorbidity prevalence including diabetes and hypertension (Table 1).

**Table 1:** Baseline characteristics of participants before PSM based on covariates.

Characteristic	Non-stroke *N* = 43,321	Stroke *N* = 1,583	*p* value
MHR	0.452 (0.235)	0.507 (0.308)	<0.001
Age, years	46.316 (16.513)	63.864 (14.298)	<0.001
Sex, *n*%			<0.001
Female	22,377 (51.55%)	808 (57.86%)	
Male	20,944 (48.45%)	775 (42.14%)	
Race, *n*%			<0.001
Mexican American	7,333 (8.00%)	159 (4.08%)	
Non-Hispanic black	8,825 (10.31%)	426 (13.83%)	
Non-Hispanic white	19,481 (69.33%)	817 (72.33%)	
Other Hispanic	3,570 (5.54%)	84 (3.11%)	
Other race	4,112 (6.82%)	97 (6.64%)	
Marital status, *n*%			<0.001
Married/living with partner	26,617 (64.78%)	820 (57.20%)	
Never married	7,673 (17.63%)	127 (7.35%)	
Widowed/divorced/separated	9,031 (17.60%)	636 (35.44%)	
Education status, *n*%			<0.001
Under high school	10,618 (15.51%)	534 (25.62%)	
High school	9,945 (23.71%)	436 (31.03%)	
Above high school	22,758 (60.78%)	613 (43.35%)	
BMI (Kg/m^2^)	28.820 (6.732)	29.862 (6.935)	<0.001
PIR	3.056 (1.636)	2.441 (1.523)	<0.001
Diabetes, *n*%			<0.001
Yes	4,760 (8.08%)	490 (28.23%)	
No	38,561 (91.92%)	1,093 (71.77%)	
Hypertension, *n*%			<0.001
Yes	14,165 (28.96%)	1,189 (72.01%)	
No	29,156 (71.04%)	394 (27.99%)	
CHD, *n*%			<0.001
Yes	1,531 (3.01%)	279 (17.83%)	
No	41,790 (96.99%)	1,304 (82.17%)	
PA, *n*%			<0.001
Yes	24,111 (49.72%)	1,106 (66.01%)	
No	19,210 (50.28%)	477 (33.99%)	
Monocytes (1,000 cells/μL)	0.564 (0.196)	0.607 (0.248)	<0.001
HDL-C (mmol/L)	1.379 (0.420)	1.350 (0.440)	0.075
AST (U/L)	24.950 (16.314)	24.262 (10.729)	0.042
ALT (U/L)	25.398 (23.034)	22.749 (14.447)	<0.001
Scr (mg/dL)	0.875 (0.360)	1.059 (0.687)	<0.001

MHR, monocyte-to-high-density lipoprotein cholesterol ratio; BMI, body mass index; PIR, ratio of family income to poverty; CHD, coronary heart disease; PA, physical activity; HDL-C, high-density lipoprotein cholesterol; AST, aspartate aminotransferase; ALT, alanine aminotransferase; Scr, serum creatinine; PSM, Propensity score matching.

To enhance the reliability of the association between MHR and stroke, PSM was implemented at 1:3 ratio. Following matching, the non-stroke cohort comprised 4,719 participants while the stroke group included 1,583 individuals. Clinically consistent with pre-matching observations, the stroke group exhibited significantly elevated MHR levels compared to non-stroke counterparts (*p* = 0.018), as detailed in Table 2. Before and after PSM, covariate balance was assessed using SMD. Prior to matching, several covariates showed significant imbalance between the groups. After matching, all included covariates achieved sufficient balance, with SMDs below 0.1, indicating minimal residual imbalance.

**Table 2:** Baseline characteristics of participants after PSM based on covariates.

Characteristic	Non-stroke *N* = 4,719	Stroke *N* = 1,583	*p* value	SMD
MHR	0.479 (0.250)	0.507 (0.308)	0.018	
Age, years	64.098 (14.007)	63.864 (14.298)	0.668	0.021
Sex, *n*%			0.933	0.003
Female	2,560 (58.03%)	808 (57.86%)		
Male	2,159 (41.97%)	775 (42.14%)		
Race, *n*%			0.948	
Mexican American	504 (3.92%)	159 (4.08%)		0.008
Non-Hispanic black	1,224 (13.50%)	426 (13.83%)		0.010
Non-Hispanic white	2,334 (72.64%)	817 (72.33%)		0.007
Other Hispanic	265 (2.94%)	84 (3.11%)		0.010
Other race-including multi-racial	392 (6.99%)	97 (6.64%)		0.014
Marital status, *n*%			0.848	
Married/living with partner	2,515 (58.14%)	820 (57.20%)		0.019
Never married	365 (6.93%)	127 (7.35%)		0.016
Widowed/divorced/separated	1,839 (34.93%)	636 (35.44%)		0.011
Education status, *n*%			0.878	
Under high school	1,650 (25.37%)	534 (25.62%)		0.006
High school	1,337 (31.89%)	436 (31.03%)		0.019
Above high school	1,732 (42.73%)	613 (43.35%)		0.012
BMI (Kg/m^2^)	30.068 (6.959)	29.862 (6.935)	0.473	0.031
PIR	2.455 (1.515)	2.441 (1.523)	0.812	0.006
Diabetes, *n*%			0.422	0.030
Yes	1,444 (26.87%)	490 (28.23%)		
No	3,275 (73.13%)	1,093 (71.77%)		
Hypertension, *n*%			0.325	0.036
Yes	3,544 (73.65%)	1,189 (72.01%)		
No	1,275 (26.35%)	394 (27.99%)		
CHD, *n*%			0.208	0.052
Yes	697 (15.83%)	279 (17.83%)		
No	4,022 (84.17%)	1,304 (82.17%)		
PA, *n*%			0.653	0.019
Yes	1,459 (34.89%)	477 (33.99%)		
No	3,260 (65.11%)	1,106 (66.01%)		
Monocytes (1,000 cells/μL)	0.594 (0.216)	0.607 (0.248)	0.163	
HDL-C (mmol/L)	1.377 (0.433)	1.350 (0.440)	0.166	
AST (U/L)	24.407 (9.928)	24.262 (10.729)	0.659	0.012
ALT (U/L)	22.710 (12.839)	22.749 (14.447)	0.944	0.004
Scr (mg/dL)	0.991 (0.614)	1.059 (0.687)	0.002	0.094

PSM was performed based on baseline covariates. The exposure variable (MHR) was not included in the matching model. Covariate balance was assessed using SMD, presented as absolute values. MHR, monocyte-to-high-density lipoprotein cholesterol ratio; BMI, body mass index; PIR, Ratio of family income to poverty; CHD, coronary heart disease; PA, physical activity; HDL-C, high-density lipoprotein cholesterol; AST, aspartate aminotransferase; ALT, alanine aminotransferase; Scr, serum creatinine; SMD, standardized mean differences; PSM, Propensity score matching.

### Association between MHR and stroke

The association between MHR and stroke susceptibility was evaluated using multivariable logistic regression models, with comprehensive results detailed in Table 3. Compared to pre-PSM analyses, the post-matching association strength demonstrated marginal attenuation while retaining statistical significance. In unadjusted models, elevated MHR exhibited significant linkage to increased stroke risk (OR = 1.462; 95% CI: 1.093–1.956), a relationship preserved in partially adjusted models (OR = 1.576; 95% CI: 1.143–2.172). This association maintained robustness in fully adjusted specifications, where each unit increment in MHR corresponded to a 51.7% elevation in stroke likelihood (OR = 1.517; 95% CI: 1.096–2.099). Subsequent quartile-based stratification revealed that participants in the highest MHR quartile (Q4) experienced 34.1% greater stroke risk compared to the lowest quartile (Q1) counterparts (OR = 1.341; 95% CI: 1.023–1.758) within fully adjusted models, demonstrating a statistically significant dose–response gradient (*P* for trend = 0.021).

**Table 3:** Association between MHR and stroke.

Variable	Model 1 OR(95%CI) *p*-value	Model 2 OR(95%CI) *p*-value	Model 3 OR(95%CI) *p*-value
Before PSM			
MHR	1.997 (1.552–2.568) <0.001	1.795 (1.344–2.396) <0.001	1.463 (1.093–1.959) 0.011
MHR quartile			
Q1 0.034–0.292	Ref.	Ref.	Ref.
Q2 0.293–0.403	1.071 (0.855–1.342) 0.546	1.088 (0.856–1.383) 0.487	1.027 (0.806–1.310) 0.827
Q3 0.403–0.550	1.173 (0.956–1.439) 0.124	1.174 (0.936–1.474) 0.164	1.022 (0.810–1.289) 0.854
Q4 > 0.551	1.619 (1.316–1.99) <0.001	1.571 (1.252–1.972) <0.001	1.268 (1.004–1.602) 0.046
P for trend	<0.001	<0.001	0.039
After PSM			
MHR	1.462 (1.093–1.956) 0.011	1.576 (1.143–2.172) 0.006	1.517 (1.096–2.099) 0.012
MHR quartile			
Q1 0.050–0.307		Ref.	Ref.
Q2 0.308–0.428	0.992 (0.771–1.278) 0.952	1.018 (0.784–1.321) 0.896	1.006 (0.776–1.305) 0.964
Q3 0.429–0.583	1.048 (0.84–1.308) 0.676	1.096 (0.861–1.396) 0.452	1.072 (0.839–1.370) 0.574
Q4 > 0.583	1.288 (1.017–1.631) 0.036	1.384 (1.057–1.811) 0.018	1.341 (1.023–1.758) 0.034
P for trend	0.020	0.010	0.021

Model 1 was the crude model with no covariates adjusted; Model 2 was adjusted for sex, age, race, BMI, marital status, education status, PIR, and PA; Model 3 was adjusted for covariates in Model 2 plus hypertension, diabetes, CHD, ALT, AST, and Scr. MHR, monocyte-to-high-density lipoprotein cholesterol ratio; BMI, body mass index; PIR, Ratio of family income to poverty; CHD, coronary heart disease; PA, physical activity; HDL-C, high-density lipoprotein cholesterol; AST, aspartate aminotransferase; ALT, alanine aminotransferase; Scr, serum creatinine; SMD, standardized mean differences; PSM, Propensity score matching; CI, confidence interval; OR, odds ratio.

### RCS analysis

**Figure 2:**
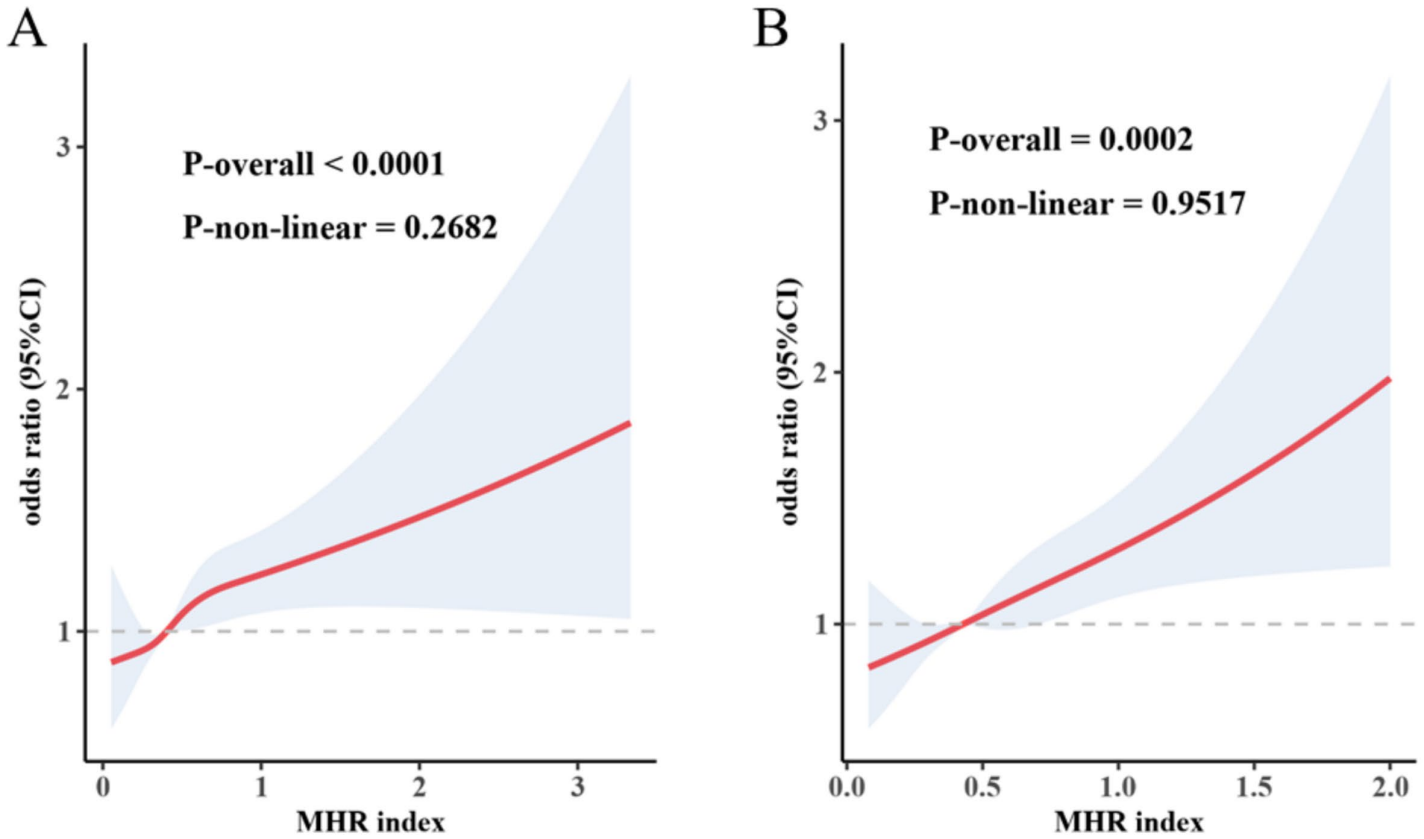
Restricted cubic spline analysis of the relationship between MHR and stroke risk before **(A)** and after **(B)** PSM. The red solid line represents the adjusted OR, and the shaded area indicates the 95% CI. The models were adjusted for the same variables as in Model 3. The reference value was set at the median of MHR. Four knots were selected for the spline, with P-overall representing the overall association and P-non-linear representing the results of the non-linear test. The sample size before PSM was *n* = 44,904, and after PSM was *n* = 6,302. MHR, monocyte-to-high-density lipoprotein cholesterol ratio; PSM, propensity score matching; CI, confidence interval; OR, odds ratio.

RCS analysis confirmed a linear dose–response pattern between MHR and stroke risk following PSM (*P*-non-linear = 0.9517), demonstrating that elevated MHR levels robustly correlate with heightened stroke susceptibility (Figure 2).

### Subgroup analysis

**Figure 3:**
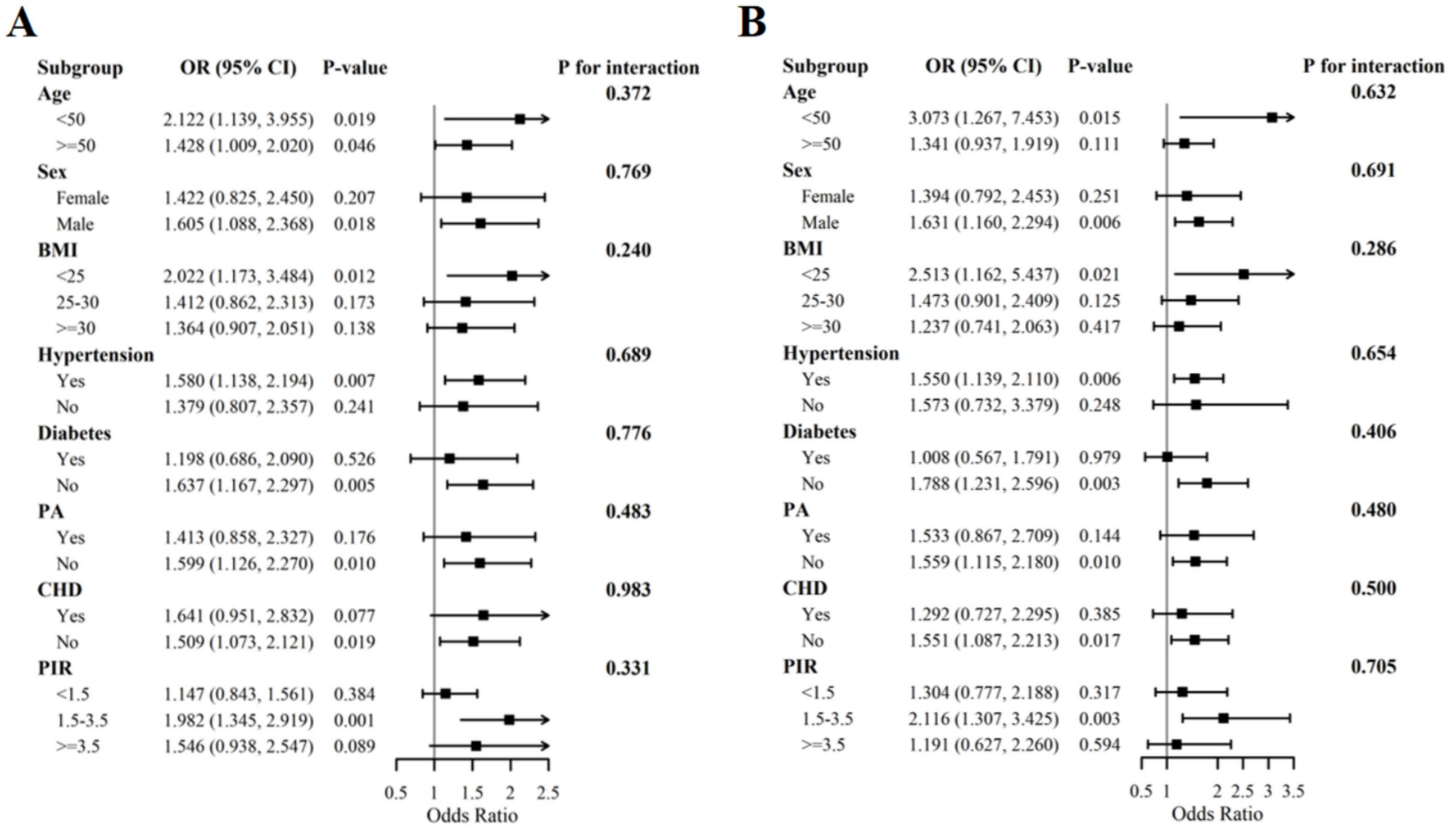
Subgroup analysis of the relationship between MHR and stroke risk before **(A)** and after **(B)** PSM. Multivariable logistic regression models were used to calculate the OR and 95% CI, adjusting for the same variables as in Model 3, but excluding the stratification variables themselves. The differences in effects between subgroups were assessed through interaction tests. The sample size before PSM was *n* = 44,904, and after PSM was *n* = 6,302. BMI, body mass index; PIR, ratio of family income to poverty; CHD, coronary heart disease; PA, physical activity; CI, confidence interval; OR, odds ratio; PSM, propensity score matching.

Subsequent stratified analyses evaluated the robustness of the positive correlation between MHR and stroke susceptibility. Within covariate-adjusted models, we examined potential interaction effects of this association across demographic and metabolic strata—including age, sex, BMI, hypertension status, and diabetes mellitus. Crucially, no statistically significant interaction terms were detected for any incorporated covariates (*P* for interaction >0.05), indicating that the MHR-stroke risk relationship operates independently of these population subgroups (Figure 3). These findings demonstrate that the positive association between elevated MHR and stroke predisposition exhibits exceptional stability and likely possesses universal applicability across diverse populations.

## Discussion

Leveraging 1999–2020 NHANES data and PSM methodology, this investigation delineated the association between MHR and stroke predisposition. The analysis revealed a statistically significant positive correlation between elevated MHR and stroke susceptibility, after adjusting for multiple covariates. RCS modeling indicated a possible linear relationship between MHR increments and cerebrovascular risk escalation. Stratified subgroup analyses suggested that the association appeared to be consistent across diverse demographic and metabolic profiles. The PSM design helped to address potential confounding biases, improving the robustness of these findings within the constraints of the study design.

Stroke pathogenesis typically involves cerebrovascular rupture or occlusion-induced localized hypoxia (15), while concurrent neuroinflammation accelerates disease progression (16). Monocytes serve as pivotal mediators in this cascade: murine ischemic stroke models reveal peak inflammatory activity at 72 h post-event (17), where monocytes function as primary sources of interleukin-1β (IL-1β), tumor necrosis factor-*α* (TNF-α), and other cytokines that disrupt blood–brain barrier integrity, exacerbating ischemia–reperfusion injury (5). Therapeutic interventions targeting monocyte phenotypic polarization demonstrate efficacy in alleviating neuroinflammation and improving stroke outcomes (18). Conversely, HDL-C operates as an inverse modulator of stroke risk through multifaceted mechanisms. The ABCA1/ABCG1 pathway mediates reverse cholesterol transport from peripheral tissues to hepatic sites, reducing vascular lipid accumulation (19) and consequently diminishing coronary artery disease and stroke susceptibility. HDL-C further attenuates oxidative stress in vascular smooth muscle cells via cholesterol efflux, exhibiting anti-inflammatory and antithrombotic properties (20). Notably, while prospective studies report an inverse correlation between physiological HDL-C concentrations and stroke incidence (21), emerging evidence identifies a paradoxical U-shaped association where extremely elevated HDL-C levels correlate with heightened stroke risk (22).

As an integrative index, the MHR simultaneously captures the pro-inflammatory burden mediated by monocytes and the anti-inflammatory and vasculoprotective properties represented by HDL-C. Consequently, MHR has demonstrated consistent clinical relevance across a range of diseases in which inflammation and endothelial dysfunction constitute central pathophysiological mechanisms. Previous studies have shown that elevated MHR is associated with an increased risk of malignant cardiac events in patients with hypertrophic cardiomyopathy (23) and has been identified as an independent predictor of mortality and major adverse cardiovascular events in patients with ST-segment elevation myocardial infarction (24). In addition, MHR has been linked to both the presence and severity of erectile dysfunction (25), a condition in which inflammation and endothelial dysfunction are considered key pathological substrates. MHR has also shown potential value in predicting the hemodynamic significance of intermediate coronary artery lesions (26).

In contrast, established inflammatory biomarkers such as the neutrophil-to-lymphocyte ratio (NLR) and the platelet-to-lymphocyte ratio (PLR) primarily reflect the intensity of systemic immune activation or inflammatory responses. Although NLR and PLR have been widely applied in risk stratification and prognostic assessment among patients with acute coronary syndromes and heart failure (27–29), these indices do not directly incorporate information related to lipid metabolism or vascular protective mechanisms. Taken together, compared with leukocyte-based ratios that solely reflect inflammatory burden, MHR integrates lipid metabolism and vascular protection on the basis of inflammatory assessment. Therefore, in studies of stroke and other atherosclerosis-related diseases, MHR may provide more disease-specific and biologically informative insights.

This study integrated monocytes and HDL-C, simultaneously capturing the dual pathological states of inflammatory activation and vascular protective decline, thereby improving the inadequacy of single indicators in disease interpretation. To enhance the validity of the results, we applied PSM in the MHR-stroke association study, aiming to minimize confounding bias as much as possible. However, this study also has certain limitations. First, due to the nature of the cross-sectional design, the temporal sequence between MHR and stroke risk cannot be clarified, thus causality cannot be inferred. Moreover, as an inherent limitation of cross-sectional studies, the timing of blood sample collection relative to stroke onset could not be determined. This heterogeneity in temporal intervals may lead to exposure misclassification, thereby constraining the interpretation of causality and the predictive value of MHR. Second, blood indicator data are derived from laboratory tests, which may have measurement errors or daily fluctuations; although we tried to use standardized laboratory data, these biases cannot be completely eliminated. Third, stroke outcomes were based on self-reported physician diagnoses, which may be affected by recall bias or outcome misclassification. However, such misclassification is more likely to be non-differential, which would generally bias effect estimates toward the null and potentially underestimate the true association between MHR and stroke. Fourth, although this study adjusted for multiple confounding factors, residual confounding from unmeasured factors (such as medication adherence, genetic susceptibility, and psychosocial stress) cannot be ruled out. While NHANES collects information on prescription medication use, including statins, detailed data on dosage, duration, and long-term adherence are incomplete across survey cycles; therefore, lipid-lowering medication use was not included in the primary analyses.

Future research still needs to rely on prospective cohort studies and interventional trials to further validate the causal association between MHR and stroke risk, and evaluate whether MHR intervention can effectively reduce stroke risk.

## Conclusion

In this nationally representative cross-sectional study, higher MHR levels were associated with an increased prevalence of stroke. Nevertheless, owing to the cross-sectional nature of the study design and the reliance on self-reported outcomes, causal inference and predictive utility cannot be established. Our findings suggest that MHR may serve as a potential population-level marker associated with stroke risk; however, prospective cohort studies are required to clarify its temporal relationship with stroke onset. In addition, the integration of Mendelian randomization analyses may help mitigate the influence of reverse causation and residual confounding, thereby strengthening causal interpretation.

## Data Availability

The data and materials in the current study are available from the corresponding author on reasonable request.

## Footnotes

1
https://wwwn.cdc.gov/nchs/nhanes/analyticguidelines.aspx
