# Supplementary material for: Association between monocyte-to-high-density lipoprotein cholesterol ratio and stroke: a propensity score matched cross-sectional study
Source: Front Neurol. 2026 Apr 16;17:1721994. doi: 10.3389/fneur.2026.1721994 (PMC13128625; doi:10.3389/fneur.2026.1721994)
Supplement: Supplementary file 1 [file Table_1.docx]

# Table S1 Covariates Included in the Analysis

| **Covariate** | **Coding / Definition** | **Inclusion Rationale** |
| --- | --- | --- |
| Age | Continuous (years) | Major demographic determinant of stroke risk |
| Sex | Male, Female | Sex-specific differences in stroke epidemiology |
| Race | NHANES-defined categories | Accounts for racial/ethnic health disparities |
| Education level | Under high school , High school, Above high school | Proxy for socioeconomic status |
| PIR | Continuous | Indicator of socioeconomic position |
| BMI | Continuous (kg/m^2^) | Established cardiometabolic risk factor |
| Marital status | Married/Living with Partner, Never married, Widowed/Divorced/  Separated | Social determinants of health |
| PA | Physical activity assessment queried whether participants engaged in any moderate-intensity recreational activities—including brisk walking, cycling, swimming, or volleyball—for ≥10 continuous minutes per session during a typical week, resulting in slight increases in respiration or heart rate (Yes / No). | Lifestyle-related stroke risk |
| Diabetes | Self-reported physician diagnosis (Yes / No) | Major metabolic risk factor |
| Hypertension | Self-reported physician diagnosis (Yes / No) | Primary stroke risk factor |
| CHD | Self-reported physician diagnosis (Yes / No) | Marker of systemic atherosclerosis |
| AST | Continuous (U/L) | Marker of hepatic function |
| ALT | Continuous (U/L) | Marker of hepatic function |
| Scr | Continuous (mg/dL) | Marker of renal function |

**Abbreviations:** BMI, body mass index; PIR, Ratio of family income to poverty; CHD, coronary heart disease; PA, physical activity; cholesterol; AST, Aspartate Aminotransferase; ALT, Alanine Aminotransferase; Scr, Serum Creatinine.
